# The prevalence of iron deficiency and anemia and their impact on survival in patients at a cardio-oncology clinic

**DOI:** 10.1186/s40959-020-00086-4

**Published:** 2020-12-02

**Authors:** E. Čiburienė, J. Čelutkienė, S. Aidietienė, G. Ščerbickaitė, A. R. Lyon

**Affiliations:** 1 Clinic of Cardiac and Vascular Diseases, Institute of Clinical Medicine, Faculty of Medicine, Vilnius University, M.K.Čiurlionis str. 21, 03101 Vilnius, Lithuania; 2 Cardio-Oncology Clinic at Royal Brompton Hospital, London, UK; 3 Imperial College London, London, UK

**Keywords:** Cardio-oncology, Cancer, Iron deficiency, Anemia, Survival

## Abstract

**Background:**

Iron deficiency (ID) and anemia are common in both heart failure (HF) and cancer patients and are associated with poor quality of life and survival. The aims of this study were (1) to evaluate the prevalence, types, and confounding factors of ID and anemia in patients referred to cardio-oncology clinic, and (2) identify the association between iron metabolism parameters and survival of cardio-oncology patients.

**Methods:**

We assessed iron, ferritin, hemoglobin concentrations, transferrin saturation (TSAT), cancer type, brain natriuretic peptide (BNP), left ventricular ejection fraction (LVEF), kidney function, cardiovascular risk factors and survival in 599 patients who were referred to cardio-oncology clinic from 2011 to 2017.

ID was defined by a TSAT < 20%, absolute iron deficiency (AID) with a serum ferritin level < 100 μg/L while serum ferritin level of ***≥*** 100 μg/L was considered as functional iron deficiency (FID) and TSAT ***≥*** 20% was considered as no ID.

**Results:**

The prevalence of ID, AID, and FID was 46, 31, and 15% of study patients, respectively. Anemia was present in approximately half (54%) of the patients with any ID.

Multivariate Cox analyses showed that male gender (HR 1.704 [1.207–2.404] *p = 0.002*); previous cancer history (HR 1.879 [1.079–3.272] *p = 0.026*); elevated BNP (HR 2.126 [1.258–3.590] *p = 0.005*); TSAT< 20% (HR 1.721 [1.214–2.439] *p = 0.002*); ferritin ***≥*** 100 μg/L (HR 2.008 [1.088–3.706] *p = 0.026*); serum iron concentration < 12 μmol/L (HR 2.292 [1.614–3.255] *p < 0.001*); FID (HR 2.538 [1.1618–3.981] *p < 0.001*) and anemia (HR 2.462 [1.734–3.495] *p < 0.001*) were significantly associated with increased risk of all-cause death.

**Conclusions:**

About half of cardio-oncology patients had anemia and iron deficiency, with the absolute type being twice as prevalent as the functional one. Patients with breast, gastrointestinal, and genitourinary cancer were affected more often. Both anemia and iron deficiency independently predicted all-cause mortality. Future studies are required to confirm ID as a risk factor and evaluate the clinical benefits of iron replacement therapy.

## Introduction

### Iron homeostasis

Iron is a fundamental element of the human body that is responsible for energy metabolism, oxygen transport and exchange, electron transfer, DNA synthesis and repair, cell growth, and proliferation [1].

Iron circulates in plasma bound to the glycoprotein transferrin, which sustains iron in a soluble form, shielding iron from developing of toxic radicals and via transferrin receptors delivers it into cells [2].

Under normal conditions, serum ferritin reflects the iron stores’ status, which correlates with the iron amount in liver biopsy samples. However, ferritin is an acute-phase protein, and infections, cancer, and liver disorders can increase serum ferritin levels, which do not correlate with real iron storage and this complicates the ID’s diagnosis [3].

### Iron deficiency in patients with cancer

Iron metabolism and regulation of homeostasis are often altered in patients with cancer. It is associated with the status of chronic disease, chronic blood loss, and nutritional deficiencies [4]. Furthermore, dividing cancer cells consume a large amount of iron for their DNA replication, growth, and metastatic process [5, 6]. The bone marrow infiltration by tumor cells or metastases and myelosuppressive chemotherapy effects are also significant for iron homeostasis [7].

Recent evidence revealed that ferritin might stimulate tumor angiogenesis, enhance tumor proliferation, and suppress the host’s immune system [8]. Elevated ferritin in cancer cells is associated with cancer progression, resistance to therapies, and worse prognosis [9]. Moreover, some cancer cells can produce ferritin, making them partly independent of external iron supply or plunder iron from surrounding cells and tissues and store it as ferritin to maintain their rapid proliferation [10].

Therefore, transferrin saturation is a more reliable marker of bioavailable iron in cancer patients [11]. ID in cancer patients is defined as TSAT < 20% with further classification of AID and FID by ferritin values: AID with exhausted iron storage (ferritin concentration < 100 μg/L) and FID with regular iron resources but restricted its availability and transport [5, 7, 12–14].

Timely diagnosis and appropriate treatment of ID are vital in cancer patients because complications are the onset or worsening of pre-existing anemia, with such symptoms as impaired exercise capacity and fatigue, deteriorated quality of life, which affects adherence to chemotherapy and may negatively influence therapeutic results [12, 15].

### Iron deficiency in patients with chronic heart failure (HF)

In recent years there has been growing interest in ID of HF patients as it may affect almost half of them, causing adverse clinical consequences. ID could be explained by the inflammatory process commonly observed in chronic HF, which creates a reduction in iron absorption and blockage in the reticuloendothelial system (RES), leading to FID. The gradual depletion of iron stores also may be caused by low iron intake, malabsorption, or blood loss, evoking AID.

It was noticed that compromised iron transport, consistent with FID, is associated with worse clinical status and increased risk of mortality in patients with chronic HF compared with patients who have decreased iron storage (AID) [16].

## Background

Advances in cancer research, diagnostics and treatment improve survival rates for many cancers. However, it varies among cancer types, stage, patients’ age and comorbidities. All influencing factors must be evaluated to increase survivability. ID and anemia are common in cancer patients and has a negative impact on the quality and expectancy of life. This study’s aims were (1) to evaluate the prevalence, types, and confounding factors of ID and anemia in patients referred to cardio-oncology clinic, and (2) identify the association between iron metabolism parameters and survival of cardio-oncology patients.

## Methods

Data were collected retrospectively in consulting referrals to the cardio-oncology clinic at Royal Brompton Hospital between 1st February 2011 and 31st May 2017 (599 patients). A median follow-up was 23.8 months.

Patients were referred from oncologists, surgeons, other cardiologists, and primary care. Common reasons for referrals to cardio-oncology clinic were (1) high baseline cardiovascular risk before an operation or cancer therapy; (2) treatment for asymptomatic or symptomatic left ventricular systolic dysfunction; (3) chemotherapy-induced vasospasm; (4) QTc prolongation and hypertension induced by vascular endothelial growth factor (VEGF) inhibitors therapy; (5) evaluation of cardiac tumors; (6) direct cardiac complications of cancer (carcinoid valvular heart disease, cardiac amyloidosis, pericardial effusion, direct invasion).

Patients with 31 types of cancer were consulted. We grouped rare cancer types into “others” (gastrointestinal stromal (GIST), mesotheliomas, neuroendocrine and nasooropharyngeal tumors).

Iron, TSAT, ferritin, hemoglobin concentrations, and red blood cell indices (mean corpuscular volume (MCV) and mean corpuscular hemoglobin (MCH)) were analyzed in all patients at admission to cardio-oncology clinic.

Reduced transferrin saturation was defined as < 20%, low iron concentration - less than 12 μmol/L.

ID was defined as TSAT < 20%, AID – TSAT < 20% and serum ferritin of < 100 μg/L, FID – TSAT< 20% and serum ferritin ***≥*** 100 μg/L. TSAT ***≥*** 20% was considered as no ID (Non-ID).

Anemia was diagnosed for a hemoglobin (Hgb) concentration < 13.0 g/L in men and < 12.0 g/L in women. Microcytic anemia was defined as mean corpuscular volume (MCV) < 84 fl and mean corpuscular hemoglobin (MCH) < 28 pg; macrocytic – MCV > 98 fl and MCH > 34 pg.

A reduced LVEF was defined as < 50%, elevated BNP was accepted as > 20 ng/L.

### Statistical analysis

We used descriptive statistics to describe distributions or variables. Continuous variables are presented as minimal (min), median or mean, maximal values (max), and standard deviation (SD). For categorical variables, frequencies and proportions (percentages) of each category or combination of categories are presented. An independent sample t-test was used to compare the values of means between two groups. To identify the significant differences between more than two groups, one-way ANOVA was performed. The independence of two categorical variables was tested using the Chi-square test. Logistic regression was used for multivariate analyses based on models, including the factors with a *p*-value ≤0.10 in univariate analyses. Odds ratios with 95% confidence intervals (CI) were reported. Risk factors for overall survival were assessed by univariate Cox regression analysis. Overall survival was defined as the time from the patient’s first visit to the cardio-oncology clinic to death from any cause. A two-tailed *p*-value of less than 0.05 considered being significant. Statistical analysis was performed using Statistical Analysis System (SAS) package version 9.2.

## Results

Data were available from 599 consecutive patients and their baseline characteristics are summarized in Table 1.

**Table 1:** Baseline characteristics of patients

Baseline Characteristics	All ***n = 599***^***1***^ (%)	ID ***n = 275*** (45.9%)	Non-ID ***n = 324*** (54.1%)	***P-value***
**Age** (mean ± SD, range)	60.4 ± 15.3 (16–93)	61.5 ± 15.2 (16–88)	59.4 ± 15.4 (18–93)	*p = 0.089*
**Sex**				***p = 0.038***
Female	363 (60.6%)	179 (65.1%)	184 (56.8%)	
**Type of visit**				*p = 0.085*
Pre-surgery/pre-chemotherapy	239 (39.9%)	122 (44.4%)	117 (36.1%)	
Current cancer treatment	243 (40.6%)	107 (38.9%)	136 (42.0%)	
Post-treatment	117 (19.5%)	46 (16.7%)	71 (21.9%)	
**Cancer location**				*p = 0.101*
Breast	181 (30.2%)	86 (31.3%)	95 (29.3%)	
Genitourinary	80 (13.4%)	32 (11.6%)	48 (14.8%)	
Sarcoma	67 (11.2%)	29 (10.5%)	38 (11.7%)	
Gastrointestinal	63 (10.5%)	33 (12.0%)	30 (9.3%)	
Thyroid	53 (8.8%)	25 (9.1%)	28 (8.6%)	
Gynecologic	50 (8.3%)	26 (9.5%)	24 (7.4%)	
Hematologic	41 (6.8%)	12 (4.4%)	29 (9.0%)	
Others	34 (5.7%)	12 (4.4%)	22 (6.8%)	
Melanoma	18 (3.0%)	12 (4.4%)	6 (1.9%)	
Lung	12 (2.0%)	8 (2.9%)	4 (1.2%)	
**Previous cancer history**	39 (6.5%)	19 (6.9%)	20 (6.2%)	*p = 0.716*
**Cardiovascular risk factors**				
Hypertension	224 (37.5%)	110 (40%)	114 (35.2%)	*p = 0.225*
Diabetes	67 (11.2%)	42 (15.3%)	25 (7.7%)	***p = 0.003***
Dyslipidaemia	79 (13.2%)	31 (11.3%)	48 (14.8%)	*p = 0.202*
Previous HF	20 (3.3%)	11 (4.0%)	9 (2.8%)	
Prior CAD	55 (9.1%)	28 (10.2%)	27 (8.3%)	
Valvular Heart Disease	17 (2.8%)	9 (3.3%)	8 (2.5%)	
Smoking	47 (7.8%)	21 (7.6%)	26 (8%)	*p = 0.966*
**Elevated BNP**	463 (77.3%)	218 (79.3%)	245 (75.6%)	*p = 0.523*
**LVEF** (mean ± SD, range, %)	57.5 ± 11.0 (20–85)	57.7 ± 10.6 (21–80)	57.4 ± 11.3 (20–85)	*p = 0.750*
LVEF< 50%	107 (17.9%)^2^	46 (16.7%)	61 (18.8%)	*p = 0.504*
**Kidney dysfunction**				***p = 0.006***
GFR < 60 ml/min/1.73 m^2^	119 (19.9%)	68 (24.7%)	51 (15.7%)	
**TSAT** (mean ± SD, range, %)	23 ± 14.1 (3.0–99.0)	13.6 ± 14.1 (3.0–19.0)	30.9 ± 14.7 (20.0–99.0)	*p < 0.001*
Median	20	14	26	
**Ferritin** (μg/L, mean ± SD, range)	188.0 ± 297.9 (6.0–1500.0)	125.7 ± 201.9 (6.0–1500.0)	241.2 ± 351.8 (11.0–1500.0)	*p < 0.001*
Ferritin < 100 μg/L	343 (57.3%)	186 (67.6%)	157 (48.4%)	
Ferritin ≥ 100 μg/L	252 (42.1%)	88 (32.0%)	164 (50.6%)	
**Serum iron** (umol/L, mean ± SD, range)	14.3 ± 7.9 (1.6–76)	9.2 ± 5.1 (1.6–76)	18.6 ± 7.2 (8.6–61.0)	***p < 0.001***
Iron < 12 umol/L	253 (42.2%)^3^	229 (83.3%)	24 (7.4%)	
**Anemia**	244 (40.7%)	144 (52.4%)	100 (30.9%)	***p < 0.001***
Normocytic	164 (27.4%)	93 (33.8%)	71 (21.9%)	
Microcytic	56 (9.4%)	47 (17.1%)	9 (2.8%)	
Macrocytic	24 (4%)	4 (1.4%)	20 (6.2%)	
**Deceased during study period**	159 (26.5%)	89 (32.4%)	70 (21.6%)	***p = 0.003***

Some data was missing: ferritin concentration in 4 patients, hemoglobin concentration in 8 patients, LVEF in 5 patients, and iron concentration in 1 patient.

*BNP* Brain natriuretic peptide, *CAD* Coronary artery disease, *GFR* Glomerular filtration rate, *HF* Heart failure, *ID* Iron deficiency, *LVEF* Left ventricular ejection fraction, *Non-ID* No iron deficiency, *SD* Standard deviation, *TSAT* Transferrin saturation.

**Fig. 1:**
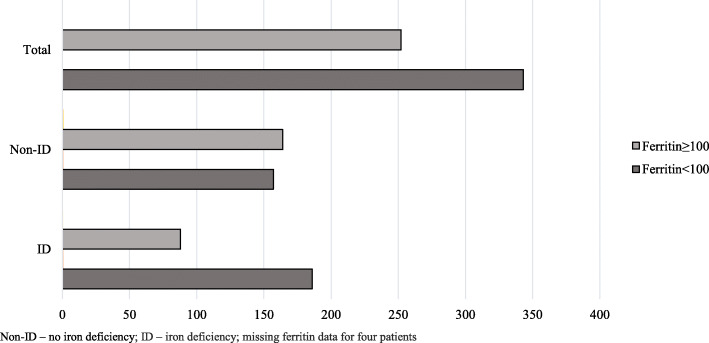
Ferritin values in patients with different iron status. Non-ID – no iron deficiency; ID – iron deficiency; missing ferritin data for four patients

Ferritin variations among study patients showed in Fig. 1.

### Confounding factors of ID

In univariate analyses gender, cancer location, elevated BNP value, DM, kidney dysfunction, and anemia were associated with an ID. In multivariate analysis taking into account all these parameters except elevated BNP and other cancer locations than breast (they appeared not statistically significant factors and were excluded from the final model), AID was independently associated with female gender (OR 2.4 [1.47; 3.84], *p < 0.01*), anemia (OR 1.9 [1.33; 2.91], *p < 0.01*), DM (OR 2.3 [1.26; 4.14], *p < 0.01*), whereas FID – with breast cancer (OR 0.4 [0.19; 0.84], *p < 0.05*) and anemia (OR 5.0 [2.98; 8.45], *p < 0.01*).

### ID subgroups analysis

Differences between iron deficiency subgroups are shown in Table 2.

**Table 2:** Differences between patients with FID and AID

Baseline Characteristics	FID ***n = 88/599*** (14.7%)	AID ***n = 186/599*** (31.1%)	***P-value***
**Age** (mean ± SD, range)	62.9 ± 13.9 (18–87)	60.9 ± 15.8 (16–86)	*p = 0.310*
**Sex**			
Female	42 (47.7%)	137 (73.3%)	***p < 0.001***
**Cancer location**			***p < 0.000***
Breast	12 (13.6%)	74 (39.6%)	***1***
Genitourinary	14 (15.9%)	18 (9.6%)	
Sarcoma	16 (18.2%)	13 (7%)	
Gastrointestinal	10 (11.4%)	23 (12.3%)	
Thyroid	6 (6.8%)	19 (10.2%)	
Gynaecologic	10 (11.4%)	16 (8.6%)	
Hematologic	6 (6.8%)	6 (3.2%)	
Others	3 (3.4%)	9 (4.8%)	
Melanoma	6 (6.8%)	6 (3.2%)	
Lung	5 (5.7%)	3 (1.6%)	
**Cardiovascular risk factors**			
Hypertension	29 (32.9%)	81 (43.5%)	*p = 0.11*
Diabetes	12 (13.6%)	29 (15.6%)	***p = 0.02***
Dyslipidaemia	6 (6.8%)	24 (12.9%)	*p = 0.142*
Smoking	21 (23.9%)	31 (16.7%)	*p = 0.6*
**Elevated BNP**	76 (89.4%)	142 (75.9%)	***p = 0.01***
**LVEF** (mean ± SD, range, %)	56.5 ± 11.3 (22–85)	58.2 ± 10.4 (21–80)	*p = 0.226*
LVEF < 50%	19 (22.1%)	24 (13%)	***p = 0.03***
**Kidney dysfunction**			
GFR < 60 ml/min/1.73 m^2^	68 (77.3%)	139 (74.3%)	*p = 0.598*
**TSAT** (mean ± SD, range, %)	13.7 ± 3.8 (4.0–19.0)	13.5 ± 4.2 (3.0–19.0)	*p = 0.707*
Median	14	14	
**Ferritin** (μg/L) (mean ± SD, range)	311.5 ± 274.5 (100.0–1468.0)	37.8 ± 23.7 (6.0–99.0)	***p < 0.001***
Median	220.5	31.5	
**Serum iron**			
(umol/L)(mean ± SD, range)	8.6 ± 7.8 (1.6–76)	9.55 ± 3.1 (1.6–17)	*p = 0.264*
**Anemia**	62 (70.4%)	82 (45.8%)	***p = 0.03***
**Deceased during the study period**	45 (51.1%)	44 (23.5%)	***p < 0.001***

*FID* Functional iron deficiency, *AID* Absolute iron deficiency, *SD* Standard deviation, *HF* Heart failure, *CAD* Coronary artery disease, *BNP* Brain natriuretic peptide, *LVEF* Left ventricular ejection fraction, *GFR* Glomerular filtration rate, *TSAT* Transferrin saturation

**Fig. 2:**
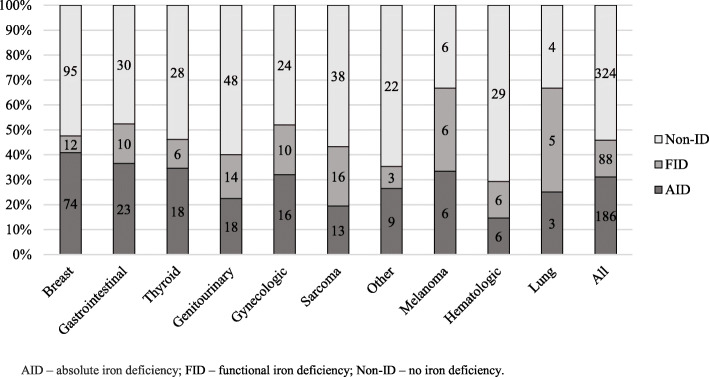
Cancer location in different iron status groups of patients. AID – absolute iron deficiency; FID – functional iron deficiency; Non-ID – no iron deficiency

AID was most often observed in patients with breast (40.9%), gastrointestinal (36.5%), and thyroid cancer (35.8%), whereas FID – in patients with lung cancer (41.7%), sarcoma (23.9%) and gynecological cancer (20%). AID and FID distribution among cancer types are presented in Fig. 2.

### Anemia in cancer patients

Almost half of all study patients had anemia. Anemia was diagnosed significantly more often in men (108 [45.8%]) than in women (136 [37.5%]) (*p = 0.032*), and men in the anemia group were older than women: 65.17 vs. 58.86 years, respectively (*p = 0.003*).

Normocytic normochromic anemia was the most frequent type in 164 (67.2%) anemic patients when microcytic hypochromic anemia was diagnosed in only 56 (22.9%).

Anemia was diagnosed in 70.4% of patients in FID group and 45.8% in AID group.

Patients with anemia more frequently had impaired kidney function (26.6% vs 15.3%, *p < 0.001*) and diabetes (53% vs 39.8%, *p = 0.054*).

**Fig. 3:**
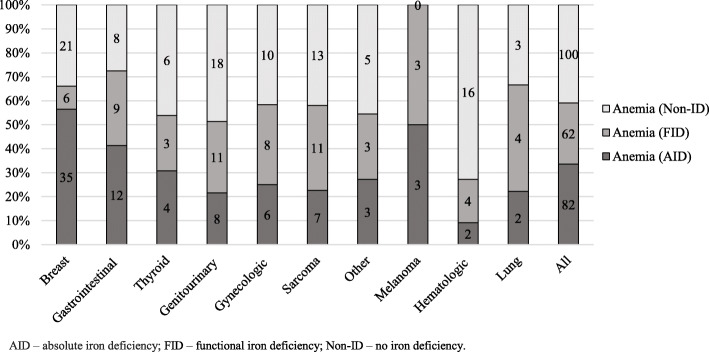
Anemia in different iron status groups of patients by cancer location. AID – absolute iron deficiency; FID – functional iron deficiency; Non-ID – no iron deficiency

**Fig. 4:**
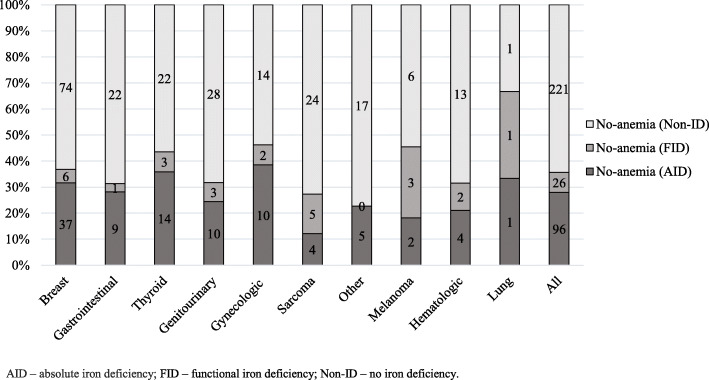
Iron status in non-anemic cancer patients. AID – absolute iron deficiency; FID – functional iron deficiency; Non-ID – no iron deficiency

Low serum iron concentration was determined in 91.6% of anemic patients (59% females), while TSAT < 20% and ferritin < 100 μg/L was found in 59 and 42.2% of anemic patients, respectively. The incidence of anemia in AID, FID, and non-ID patients with different cancer locations showed in Fig. 3. Iron status among patients without anemia is presented in Fig. 4.

### Prognostic impact of ID in cancer patients

During the study period, 159 patients died, more men than women (35.6% vs. 20.7%).

Deceased patients were older and almost 90% of them had elevated BNP; their ferritin levels were higher, but they had lower mean serum iron concentration.

Patients in the ID group died more frequently than in the non-ID *(p = 0.003,* see Table 1*)*; the risk of death in patients with FID was almost four times higher than in patients with AID (OR 3.797 [2.319; 6.249], *p < 0.01*).

Deceased patient characteristics are presented in Table 3.

**Table 3:** Deceased patients’ characteristics

	Deceased ***n = 159*** (26.5%)	Alive ***n = 440*** (73.5%)	***P-value***
**Age** (mean ± SD, range)	63.9 ± 14.2 (21–93)	59.1 ± 15.3 (16.5–90)	***p < 0.001***
**Gender**			
Female	75 (47.2%)	288 (65.5%)	***p < 0.001***
**Cancer location**			***p < 0.001***
Genitourinary (GU)	27 (17.0%)	53 (12%)	
Sarcoma	27 (17.0%)	40 (9%)	
Gastrointestinal (GI)	21 (13.2%)	42 (9.5%)	
Gynecologic (Gyn)	21 (13.2%)	29 (6.6%)	
Breast	20 (12.6%)	161 (36.6%)	
Thyroid	11 (6.9%)	42 (9.5%)	
Lung	8 (5.0%)	4 (0.9%)	
Hematologic (Hem)	8 (5.0%)	33 (7.5%)	
Melanoma	6 (3.8%)	12 (2.7%)	
Other	10 (6.3%)	24 (5.5%)	
**BNP elevation**	139 (89.1%)	324 (74.1%)	***p < 0.001***
**LV dysfunction**			
LVEF < 50%	33 (20.8%)	74 (16.8%)	*p = 0.267*
**GFR < 60 ml/min/1.73m**^**2**^	39 (24.5%)	80 (18.2%)	*p = 0.086*
**TSAT** (mean ± SD, range, %),	21.9 ± 16.3 (3.0–99.0)	23.3 ± 13.2 (4.0–94.0)	*p = 0.313*
Median	20	20	
**Ferritin** (μg/L) (mean ± SD, range)	245 ± 319.4 (11.0–1500)	167.4 ± 287.3 (6–1500)	***p = 0.005***
Ferritin < 100 μg/L	71 (44.6%)	272 (61.8%)	
Ferritin ***≥*** 100 μg/L	87 (54.7%)	165 (37.5%)	
**Serum iron** (umol/L)(mean ± SD.range)	12.6 ± 8.2 (1.6–54.2)	14.9 ± 7.7 (1.7–76)	***p = 0.002***
Iron < 12 umol/L	93 (58.5%)	159 (36.1%)	
**Anemia**	94 (59.1%)	150 (34.1%)	*p = 0.740*

Some data was missing: there was no data about ferritin concentration of 1 and BNP of 3 deceased patients

**Fig. 5:**
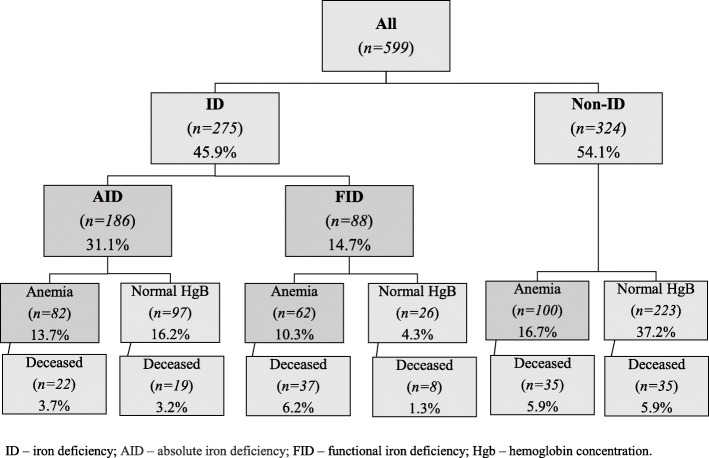
Iron metabolism parameters and their relation to survival. ID – iron deficiency; AID – absolute iron deficiency; FID – functional iron deficiency; Hgb – hemoglobin concentration

The relation between iron status and survival showed in Fig. 5.

The prognostic impact of different factors is presented in Table 4. Using univariate Cox regression model we found that male gender, cancer location except hematologic and other type of cancer (gastrointestinal HR 2.991 [1.570–5.699] *p < 0.001*; genitourinary HR 2.530 [1.359–4.709] *p = 0.003*; gynecological 4.426 [2.300–8.517] *p < 0.001*; hematological 1.640 [0.689–3.903] *p = 0.263*; lung HR 8.354 [3.116–22.397] *p < 0.001*; melanoma HR 3.176 [1.080–9.341] *p = 0.036*; other HR 2.112 [0.888–5.024] *p = 0.091*; sarcoma HR 3.760 [2.021–6.997] *p < 0.001*; thyroid HR 2.152 [1.023–4.526] *p = 0.043*), previous cancer history, elevated BNP (> 20 ng/L), TSAT < 20%, ferritin ***≥*** 100 μg/L, serum iron concentration < 12 umol/L and anemia were significantly associated with increased risk of all-cause death.

**Table 4:** Factors affecting survival

Factor	Category	HR (95% CI)	***P-value***
**Gender**	Male	1.704 (1.207–2.404)	***p = 0.002***
**Age**		1.016 (1.004–1.028)	***p = 0.011***
**Previous cancer history**	Yes	1.879 (1.079–3.272)	***p = 0.026***
**BNP**	Elevated	2.126 (1.258–3.590)	***p = 0.005***
**LVEF**	< 50%	1.101 (0.721–1.683)	*p = 0.655*
**GFR**	< 60 ml/min/1.73 m^2^	1.255 (0.838–1.879)	*p = 0.270*
**TSAT**	< 20%	1.721 (1.214–2.439)	***p = 0.002***
**Ferritin**	20–100 μg/L	1.004 (0.532–1.892)	*p = 0.991*
	***≥***100 μg/L	2.008 (1.088–3.706)	***p = 0.026***
**ID**	FID	2.538 (1.162–3.981)	***p < 0.001***
**Serum iron**	< 12 umol/L	2.292 (1.614–3.255)	***p < 0.001***
**Anemia**	Yes	2.462 (1.734–3.495)	***p < 0.001***

*HR* Hazard ratio, *BNP* Brain natriuretic peptide, *LVEF* Left ventricular ejection fraction, *GFR* Glomerular filtration rate, *TSAT* Transferrin saturation, *ID* Iron deficiency, *FID* Functional iron deficiency

## Discussion

These are the first data on prevalence and patterns of iron metabolism disorders in cardio-oncology patients, showing an independent association of ID and all-cause mortality. The present study’s critical findings are that ID is ubiquitous in cardio-oncology patients, AID is more prevalent than FID, and the iron deficit is a significant factor associated with prognosis and clinical outcomes in this population. However, ID often remains undiagnosed and untreated properly in cancer patients because the assessment of iron status under inflammatory conditions is complicated and demands more attention to iron homeostatic mechanisms when intravenous iron administration alone or in combination with erythropoiesis-stimulating agents is safe and more effective than oral iron forms and also decreases the requirement for transfusion [17, 18].

Our results revealed a high prevalence of ID (46%) in cardio-oncology patients. ID is always caused by a disbalance between iron availability and requirement. This balance is strictly regulated but could be disturbed in cancer patients by a combination of a chronic inflammatory process, alimentary deficiencies, blood loss, increased iron consumption by cancer cells, bone marrow infiltration, and chemotherapy-caused myelosuppression [4–7]. The high prevalence of ID in such cancer types as gastrointestinal, genitourinary, gynecological, lung may be explained by the increased risk of bleeding. Although, some cancer cells can increase the intracellular iron stores for their proliferative and metastatic purposes, thus reducing iron store levels. Moreover, ID is associated with a more advanced cancer stage and a more malignant phenotype [5, 19].

FID is reported as the dominant iron deficiency mechanism in oncology patients triggered by the inflammatory process associated with cancer or its treatment [4, 16]. The elevated ferritin values are associated with acute infection, kidney dysfunction, liver disorders, and more aggressive disease [3]. Although FID is associated with worse clinical status and increased risk of mortality in these patients, we found that in cardio-oncology patients’ population AID proportions were twice as high as FID, similar to patients with chronic HF but without cancer [16, 20]. However, ID, FID, and anemia were more reliable predictors of mortality than elevated BNP in our study. Surprisingly, cardiovascular risk factors (AH, diabetes, dyslipidemia, smoking, and previous cardiac disease) showed no statistically significant effect on mortality among our patients.

It is well known that anemia is widespread (28–45%) and strongly associated with higher mortality in cancer patients [21, 22]. We determined that almost half of our patients with ID (41%) had anemia, and more than half (59%) died during the study period. The prevalence of anemia and mortality was higher among FID.

We found out that diabetes and kidney dysfunction was significantly associated with ID. In case of diabetes, it could be explained by chronic inflammation and kidney dysfunction as a complication. On the other hand, reduced iron stores are associated with increased hemoglobin A1C concentration, leading to a false-positive diabetes diagnosis [23]. In chronic kidney disease ID is often caused by reduced hepcidin excretion by the kidneys, increased blood loss, and treatment with erythropoietin-stimulating agents [3].

In summary, iron status should be assessed in all cancer and HF patients not only for the reason to prevent or correct anemia and improve patient’s symptoms but to potentially reduce mortality and contribute to improving survival rates in this complex group of patients.

Our results differ from other ID research data in cancer patients because patients referred to cardio-oncology clinic were older, had concomitant cardiovascular disease or its risk factors, and other severe comorbidities.

### Study limitations

Presented data were obtained retrospectively from only one blood sample at the admittance to the cardio-oncology clinic, and there was impossible to take into account random fluctuations of iron parameters. Some data was missing. A wide variety of different types of cancer were included in this study, but some cancer types were rare for statistically significant results. In multivariate analysis, such cancer types as lung, melanoma, thyroid, and hematologic were grouped with “Others” due to small groups’ size.

Measurements of TSAT have some limitations. In inflammatory diseases, the expression of transferrin receptors is reduced, resulting in a falsely high transferrin concentration, which decreases the TSAT even when the circulating iron level is stable [13]. However, malnutrition and chronic disease may diminish the synthesis of transferrin, which would raise TSAT. There are also significant (17–70%) diurnal fluctuations in TSAT concentration that may challenge interpreting this value [11].

For a more accurate evaluation of ID subgroups, C-reactive protein, hepcidin, and soluble transferrin receptors concentration would be helpful, but these analyses were unavailable [24, 25].

Unfortunately, there is no data about iron therapy in these patients, which may have had a prognostic impact.

## Conclusion

Iron deficiency and anemia are common in cancer patients referred to a cardio-oncology service. The highest ID rates were in patients with breast, gastrointestinal, genitourinary cancer, and sarcoma. AID was detected more often than FID. FID more frequently was associated with breast cancer, anemia, and worse survival prognosis, whereas AID – with female gender, anemia, and diabetes. Low transferrin saturation, ferritin ***≥*** 100 μg/L, iron level < 12 μmol/L, functional iron deficiency, and anemia independently predicted all-cause death.

Future studies are required to confirm ID as a risk factor and evaluate iron replacement therapy’s clinical benefits.

## Data Availability

All data and materials are available upon request.

## Abbreviations

AH: Arterial hypertension
AID: Absolute iron deficiency
BNP: Brain natriuretic peptide
CAD: Coronary artery disease
CV: Cardiovascular
DM: Diabetes mellitus
DNA: Deoxyribonucleic acid
FID: Functional iron deficiency
GI: Gastrointestinal cancer
GFR: Glomerular filtration rate
Gyn: Gynecological cancer
GU: Genitourinary cancer
Hem: Hematological cancer
HF: Heart failure
Hgb: Hemoglobin
HR: Hazard ratio
ID: Iron deficiency
LVEF: Left ventricular ejection fraction
MCH: Mean corpuscular hemoglobin
MCV: Mean corpuscular volume
Non-ID: No iron deficiency
OR: Odds ratio
RES: Reticuloendothelial system
SAS: Statistical Analysis System
SD: Standard deviation
TSAT: Transferrin saturation
VEGF: Vascular endothelial growth factor
Vs.: Versus
